# Miglitol increases energy expenditure by upregulating uncoupling protein 1 of brown adipose tissue and reduces obesity in dietary-induced obese mice

**DOI:** 10.1186/1743-7075-11-14

**Published:** 2014-03-26

**Authors:** Satoru Sugimoto, Hisakazu Nakajima, Kazuki Kodo, Jun Mori, Kensuke Matsuo, Kitaro Kosaka, Wataru Aoi, Kanji Yoshimoto, Hiroshi Ikegaya, Hajime Hosoi

**Affiliations:** 1 Department of Pediatrics, Graduate School of Medical Science, Kyoto Prefectural University of Medicine, 465-Kajiicho, Hirokoji-Kawaramachi, Kamigyo-ku, Kyoto 602-8566, Japan; 2 Laboratory of Health Science, Graduate School of Life and Environmental Sciences, Kyoto Prefectural University, Kyoto, Japan; 3 Department of Forensic Medicine, Graduate School of Medical Science, Kyoto Prefectural University of Medicine, Kyoto, Japan

**Keywords:** Miglitol, Alpha-glucosidase inhibitor, Obesity, Oxygen consumption, Brown adipose tissue, Uncoupling protein 1, β3-adrenergic signaling

## Abstract

**Background:**

Miglitol is an oral anti-diabetic drug that acts by inhibiting carbohydrate absorption in the small intestine. Recent studies have shown that miglitol reduces obesity in humans and rodents. However, its mechanisms have remained unclear. The purpose of this study was to determine whether miglitol generates heat by activating uncoupling protein 1 (UCP1), an enzyme involved in thermogenesis, in brown adipose tissue (BAT) in mice.

**Methods:**

Four-week-old male C57BL/6 J mice were fed a high-fat diet alone (HF) or a high fat diet plus miglitol (HFM). Oxygen consumption (VO_2_) was used to estimate metabolic rate. A thermal imaging camera was used to quantify heat generation from interscapular brown adipose tissue. We analyzed the protein and gene expressions of UCP1 and the expressions of four proteins related to β3-adrenergic signaling in the pathway activating UCP1 (protein kinase A (PKA), hormone-sensitive lipase (HSL), p38 α mitogen-activated protein kinase (p38αMAPK) and peroxisome proliferator-activated receptor gamma coactivator 1α (PGC1α)).

**Results:**

At 8 weeks, body weight, epididymal and subcutaneous white adipose tissue and the HOMA-R value of the HFM mice were significantly less than those of the HF mice. Food intake was not different between the HF and HFM mice. VO_2_ and BAT temperature were significantly higher in the HFM mice. Miglitol significantly enhanced the gene and protein expressions of UCP1 and the expressions of proteins related to β3-adrenergic signaling.

**Conclusions:**

Miglitol’s anti-obesity effect was attributed to increased energy expenditure by upregulating UCP1 in BAT (i.e., by thermogenesis) and to enhancement of β3-adrenergic signaling in BAT.

## Background

Obesity develops from an imbalance between energy intake and energy expenditure [1]. At present, only medicines that inhibit energy intake, such as appetite suppressants and lipid absorption inhibitors, are authorized as anti-obesity drugs by the American Food and Drug Administration (FDA). Enhancement of energy expenditure has emerged as a potential and attractive strategy for treating or preventing obesity. Whereas white adipose tissue acts to store surplus energy, brown adipose tissue (BAT) expends energy by heat production through uncoupling protein 1 (UCP1) in its mitochondria [2]. In general, it has been believed that humans lose BAT shortly after infancy. However, recent studies using positron emission tomography/computed tomography (PET/CT) have shown that adult humans still possess functional BAT. BAT has received much attention as a target of obesity treatment [3-7].

Miglitol is an alpha-glucosidase inhibitor (αGI) and is commonly used as an anti-diabetic drug [8]. In diabetic subjects, miglitol blunts postprandial hyperglycemia by inhibiting alpha-glucosidase in the small intestine and prolongs carbohydrate absorption. Recently, miglitol has been reported to have an anti-obesity effect [9-11]. However, its mechanisms are not clear. Here we examined the effect of miglitol on UCP1, an enzyme involved in thermogenesis, in BAT. Our results show that miglitol increased energy expenditure, reduced obesity and enhanced β3-adrenergic signaling and upregulation of UCP1 in BAT. These data provide further support for miglitol as an anti-obesity agent and clarify its mechanism of action.

## Methods

### Animals and diets

Four-week-old male C57BL/6 J mice were purchased from CLEA Japan (Tokyo, Japan). Four diets were prepared: normal chow (CLEA Rodent diet CE-2: 12% of calories from fat, 59.1% of calories from carbohydrate, 28.8% of calories from protein), a high fat diet (HFD) (Clea High fat diet 32: 56.7% of calories from fat, 23.1% of calories from carbohydrate, 20% of calories from protein), normal chow containing 0.008% miglitol and HFD containing 0.008% miglitol. A previous study of miglitol in mice [11] used a diet containing 0.08% miglitol. We chose to use a lower dose because it was closer to the dose used in clinical medicine. Mice were divided into 4 groups: a control group (NC), which was fed normal chow, a normal chow plus miglitol (NCM) group, which was fed the normal chow plus miglitol, a high fat (HF) group, which was fed the HFD, and a high fat plus miglitol (HFM) group, which was fed the HFD plus miglitol. The mice were kept in a temperature-controlled room (23°C) on a 12 h light/dark cycle (lights on 07:00 h; off 19:00 h) with free access to food and water. Individual food intake and body weight gain were monitored once a week. At 8 weeks, mice were fasted overnight and anaesthetized with sodium pentobarbital (50 mg/kg, i.p.) and blood was obtained by cardiopuncture. Plasma was separated by centrifugation at 4°C and stored at -80°C until assayed. The epididymal and subcutaneous white adipose tissues were dissected and weighed. Interscapular brown adipose tissue and liver were immediately dissected, frozen in liquid nitrogen and stored at -80°C until further analysis. All animal experiments and care procedures were conducted in conformity with the Guidelines of the Animal Care and Use Committee of Kyoto Prefectural University of Medicine.

### Plasma parameters

Blood glucose was determined with a compact glucose analyzer Antsense II (Horiba, Kyoto, Japan). Plasma triglyceride (TG) and total cholesterol (T-Cho) levels were measured with reagents from Wako (Osaka, Japan). Plasma insulin level was measured by an ELISA kit (Morinaga Institute of Biological Science, Kanagawa, Japan). Plasma active glucagon-like peptide 1 (GLP1) levels were measured with an ELISA kit (Shibayagi, Gunma, Japan). All of the assays were performed according to the manufacturer’s instructions. Serum concentration of miglitol was measured by liquid chromatography - tandem mass spectrometry (LC/MS/MS).

### Oxygen consumption

Oxygen consumption (VO_2_) was measured with an O_2_/CO_2_ metabolism-measuring system (model MK-5000, Muromachi-Kikai, Tokyo, Japan), which consists of two independent 560-ml chambers (for measuring two animals simultaneously), a suction pump and a computer for data analysis [12]. The mice were placed in the chambers at 23°C and acclimated for more than two hours. Every three minutes, the pump draws air from one of the chambers for one minute at rate of a 650 ml/min to measure O_2_ concentration. Oxygen consumption (VO_2_) was calculated as [Oa-Oc]v m^-1^ t^-1^, where Oa is the atmospheric oxygen concentration (%) that flows into the chamber, Oc is the oxygen concentration in the chamber (%), v is the flow rate (650 milliliters/min), m is the mass of the mouse in kg and t is the time in hours [13].

### Interscapular temperature

Mice were fasted for 6 hours and anaesthetized (sodium pentobarbital, 30 mg/kg, i.p.). Interscapular temperature surrounding BAT was recorded with a thermal imaging camera (FLIR i3, FLIR Systems, Tokyo, Japan) and analyzed with FLIR QuickReport software.

### Histology

BAT was fixed in 10% buffered formalin. Sections (5 μm) were stained with hematoxylin and eosin. Slides were examined and photomicrographs taken under the same exposure and magnification. Lipid droplets in cells of BAT were quantified as previously described [14]. One tissue section from each mouse was measured under blinded conditions by one investigator (S.S.) counting the number of nuclei surrounded by four or more lipid vacuoles/cell in two randomly chosen areas (1600 μm^2^) of each section, and averaging the results.

### Western blot analysis

BAT was lysed with radioimmunoprecipitation assay (RIPA) lysis buffer (Nacalai Tesque, Kyoto, Japan). Homogenates were centrifuged at 10,000 × g for 10 min at 4°C and supernatants were collected. Protein concentrations were determined with a Bio-Rad protein assay kit (Bio-Rad, Tokyo, Japan). Tissue proteins were resolved on 10% polyacrylamide gels in the presence of sodium dodecyl sulfate, transferred electrophoretically to polyvinylidene difluoride membranes, and blocked by Blocking One (Nacalai Tesque). The primary and secondary antibodies were diluted with Can Get Signal (Toyobo, Osaka, Japan). The membrane was incubated with primary antibodies against proliferator-activated receptor gamma coactivator 1α (PGC1α) (1:10,000) (Abcam, Tokyo, Japan), UCP1 (1:15,000) (Abcam), β3-adrenergic receptor (β3AR) (1:10,000) (Abcam), protein kinase A (PKA) (1:5,000) (Santa Cruz Biotechnology, Santa Cruz, CA), phosphorylated-protein kinase A (*p*PKA) (1:5,000) (Santa Cruz Biotechnology, Santa Cruz, CA), hormone-sensitive lipase (HSL) (1:10,000) (Cell Signaling Technology, Tokyo, Japan), carnitine palmitoyltransferase1 (CPT1) (1:5,000) (Lifespan Biosciences, Seattle, WA), p38α mitogen-activated protein kinase (p38αMAPK) (1:5,000) (Cell Signaling Technology), and β-actin (1:5,000) (Cell Signaling Technology). Secondary antibody consisted of a 1:15,000 dilution of HRP-conjugated donkey anti-rabbit IgG (for PGC1α, UCP1, β3AR, PKA, *p*PKA, HSL, CPT1, p38αMAPK) (GE Healthcare, Tokyo, Japan) or HRP-conjugated sheep anti-mouse IgG (for β-actin) (GE Healthcare). The immunocomplexes were detected using an enhanced HRP-luminol chemiluminescence system (ECL prime) (GE Healthcare) and subjected to autoradiography (New Amersham Hyperfilm) (GE Healthcare). Signals on the immunoblot were quantified using the NIH Image computer program (NIH, Bethesda, MD, version 1.45). To compare the results for protein expression, we assigned a value of 1 to expression in BAT from control mice.

### Cyclic AMP (cAMP) assay

The selective β3-adrenergic receptor agonist CL316,243 (Sigma, Tokyo, Japan) (2 mg/kg/body weight) and saline was given by intraperitoneal injection 6 h before the end of the experiment. The amount of cAMP in BAT was measured by a cAMP assay kit (R&D Systems, Minneapolis, MN) according to the manufacturer’s instructions.

### Quantitative real-time PCR

Total RNA from BAT and liver were isolated using a NucleoSpin RNA II kit (Macherey-Nagel, Düren, Germany). Template cDNA synthesized from 500 ng total RNA with random hexamer primers was used as the template for each reaction with a SuperScript First-Strand Synthesis System (Invitrogen Life Technology, Osaka, Japan). Quantitative real-time PCR (qRT-PCR) was performed using a SYBR Green master mix (Takara, Shiga, Japan) with 10 μM of each primer in an AB 7300 Real-Time PCR System (Applied Biosystems, Tokyo, Japan). Amplification was performed with the following protocol: 40 cycles (5 sec at 95°C and 31 sec at 60°C) after an initial activation step for 30 sec at 95°C. Primer sequences were shown as follows: β-actin (BAT), forward primer: 5′-GAAATCGTGCGTGACATCAAAG-3′, reverse primer: 5′-TGTAGTTTCATGATGCCACAG-3′; β-actin (liver), forward primer: 5′-GGCTGTATTCCCCTCCATCG-3′, reverse primer: 5′-CCAGTTGGTAACAATGCCATGT-3′; PGC1α, forward primer: 5′-TGAACGCACCTTAAGTGTGGAA-3′, reverse primer: 5′- GGGTTATCTTGGTTGGCTTTATGA-3′; UCP1, forward primer: 5′- AGGCTTCCAGTACCATTAGGT -3′, reverse primer: 5′-CTGAGTGAGGCAAAGCTGATTT-3′; CPT1, forward primer: 5′-CCAATCATCTGGGTGCTGG-3′, reverse primer: 5′-AAGAGACCCCGTAGCCATCA-3′; glucokinase **(**GK), forward primer: 5′-CAACTGGACCAAGGGCTTCAA-3′, reverse primer: 5′-TGTGGCCACCGTGTCATTC-3′. β-actin was chosen as an internal standard.

### Statistical analysis

Data are shown as means ± SEM. Single-group data were assessed using Student’s t-test. Repeated measurements of analysis of variance (ANOVA) with Tukey-Kramer post hoc comparisons were performed for multiple comparisons. P values less than 0.05 were considered statistically significant.

## Results

### Miglitol reduced body weight gain and increased energy expenditure in high fat diet-induced obese mice

**Figure 1:**
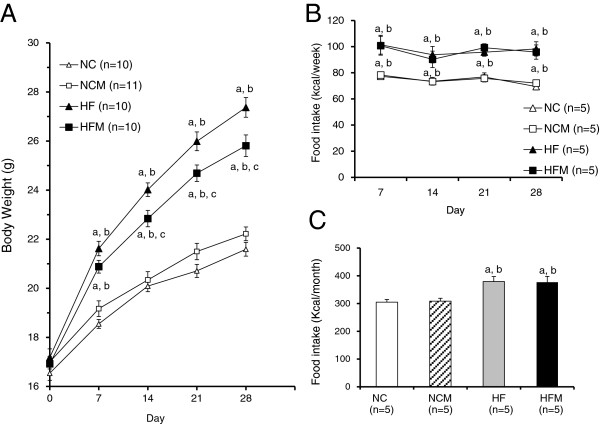
**Effect of miglitol on body weight gain. A**: Body weight change. **B**: Weekly food intake. **C**: Total food intake. Values are means ± SE for 5–11 mice. ^a^P < 0.05, vs mice fed normal chow diet (NC). ^b^P < 0.05, vs mice fed normal chow diet plus miglitol (NCM). ^c^P < 0.05, vs mice fed high-fat diet alone (HF).

**Figure 2:**
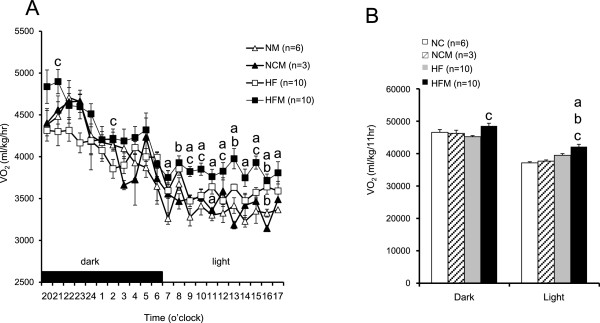
**Effect of miglitol on energy expenditure. A**: 22-h Oxygen consumption. **B**: Oxygen consumption in dark and light phases. Values are means ± SE for 3–10 mice. ^a^P < 0.05, vs mice fed normal chow diet (NC). ^b^P < 0.05, vs mice fed normal chow diet plus miglitol (NCM). ^c^P < 0.05, vs mice fed high-fat diet alone (HF).

**Figure 3:**
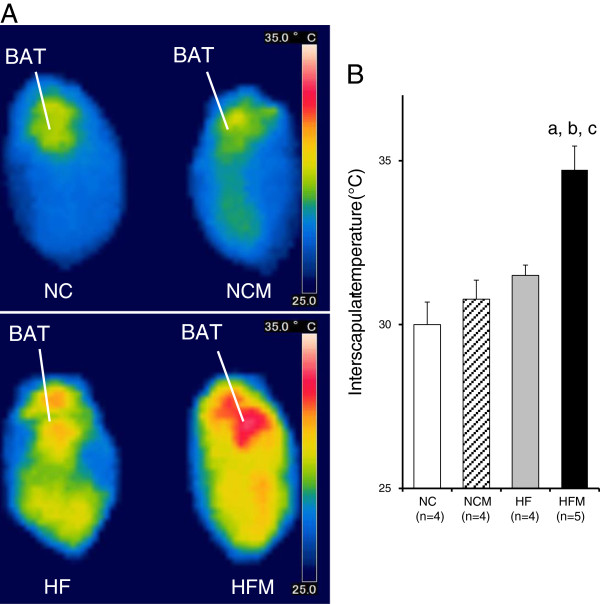
**Effect of miglitol on BAT temperature. A** shows representative infrared thermal image of normal chow diet mice (NC), miglitol-treated normal chow diet mice (NCM), high-fat diet mice (HF) and miglitol-treated high-fat diet mice (HFM). **B**: Interscapular temperature was measured. Values are means ± SE for 4–5 mice. ^a^P < 0.05, vs mice fed normal chow diet (NC). ^b^P < 0.05, vs mice fed normal chow diet plus miglitol (NCM). ^c^P < 0.05, vs mice fed high-fat diet alone (HF).

The body weight of mice fed a high fat diet (HF mice) (27.3 ± 0.4 g at 8 weeks) was significantly greater than that of mice fed normal chow (p < 0.05) (control mice) (21.5 ± 0.2 g). The body weight of mice fed the high fat diet plus miglitol (HFM mice) (25.8 ± 0.4 g) was significantly less than that of HF mice (p < 0.05) (Table 1, Figure 1A) even though the two groups consumed the same amount of food energy (Figure 1B, C). On the other hand, miglitol did not affect the body weight under the condition of normal chow (Table 1, Figure 1A). The miglitol-treated mice did not manifest any of the common adverse effects of miglitol, such as gastrointestinal abnormalities, diarrhea or anorexia. Oxygen consumption (VO_2_), an indirect measurement of metabolism, was significantly increased in HFM mice compared to HF mice in both the dark and light phases (p < 0.05) (Figure 2). By contrast, VO_2_ was not different between NC and NCM mice. Interscapular BAT temperature in HFM mice (34.7 ± 0.7°C) was significantly higher (p < 0.05) than that in HF mice (31.5 ± 0.3°C) and was significantly higher (p < 0.05) than the temperatures in NC mice (30.0 ± 0.6°C) and NCM mice (30.7 ± 0.5°C) (Figure 3).

**Table 1:** Metabolic parameters at 8-weeks-old mice

	**n**	**NC**	**NCM**	**HF**	**HFM**
Body weight (g)	10-11	21.5 ± 0.2	22.2 ± 0.2	27.3 ± 0.4 ^a, b^	25.8 ± 0.4 ^a, b, c^
Glucose (mg/dl)	5	118 ± 6.6	129 ± 15.4	282 ± 4.1 ^a, b^	240 ± 9.1 ^a, b, c^
Total cholesterol (mg/dl)	5-7	62 ± 4.7	68 ± 4.9	150 ± 7.2 ^a, b^	141 ± 4.2 ^a, b^
Triglyceride (mg/dl)	6	42 ± 8.4	45 ± 5.7	42 ± 4.9	46 ± 5.2
Insulin (μU/ml)	5	5.3 ± 1.2	3.4 ± 0.5	12.3 ± 1.9 ^a, b^	6.7 ± 1.2
HOMA-R	5	1.4 ± 0.3	1.1 ± 0.3	8.4 ± 1.3 ^a, b^	4.0 ± 0.7 ^a, b, c^
Weight of epididymal white adipose tissue (g)	9-14	0.27 ± 0.02	0.28 ± 0.01	1.1 ± 0.08 ^a, b^	0.85 ± 0.04 ^a, b, c^
Weight of subcutaneous white adipose tissue (g)	6	0.3 ± 0.03	Not measured	1.5 ± 0.15 ^a^	0.98 ± 0.12 ^a, c^
Active glucagon-like peptide1 (pg/ml)	8-9	54.8 ± 7.9	61.1 ± 4.9	66 ± 7.5	76.9 ± 14.4
Concentration of miglitol (μmol/L)	3-4	Not measured	0.06 ± 0.02	Not measured	0.26 ± 0.13

Values are means ± SE for 3–14 mice. Blood samples were collected under fasting conditions. ^a^P < 0.05, vs mice fed normal chow diet (NC). ^b^P < 0.05, vs mice fed normal chow diet plus miglitol (NCM). ^c^P < 0.05, vs mice fed high-fat diet alone (HF).

### Miglitol ameliorated insulin resistance in high fat diet-induced obese mice

Blood glucose and plasma T-Cho levels were significantly higher in HF mice than in NC and NCM mice. Blood glucose levels were significantly lower in HFM mice than in HF mice. Plasma TG did not differ in the four groups. The HOMA-R value, an index of insulin resistance, was significantly elevated in HF mice and significantly lowered by miglitol (control 1.4 ± 0.3 vs. NM 1.1 ± 0.3 vs. HF 8.4 ± 1.3 vs. HFM 4.0 ± 0.7) (CC or NC vs. HF, p < 0.05; HF vs. HFM, p < 0.05). The masses of epididymal and subcutaneous white adipose were lower in the HFM mice than in the HF mice (Table 1).

Miglitol enhances the secretion of active glucagon-like-peptide1 (GLP1) in obese humans (see “Discussion” section). Because GLP1 decreases food intake, many clinicians attribute miglitol’s anti-obesity effect to suppression of food intake. However, the active GLP1 level did not differ between HF and HFM mice (Table 1).

### Miglitol decreased the number of lipid droplets in BAT cells of HF mice

**Figure 4:**
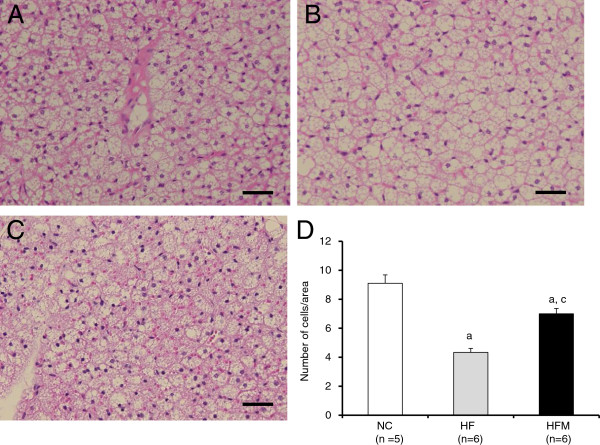
**Effect of miglitol on the number of lipid droplets in BAT cells.** Histology of BAT was examined by HE staining (Scale bar = 40 μm). **A**, **B** and **C** show the representative histology of normal chow diet mice, high-fat diet mice and miglitol-treated high-fat diet mice, respectively. **D**: The number of cells/area was counted. Values are means ± SE for 5–6 mice. ^a^P < 0.05, vs mice fed normal chow diet (NC). ^c^P < 0.05, vs mice fed high-fat diet alone (HF).

To evaluate the degree of lipolysis, we investigated the microscopic appearance of BAT. The HFD increased the number of lipid droplets in cells of BAT, while miglitol significantly decreased the number of lipid droplets in cells of BAT (NC 9.1 ± 0.5 vs. HF 4.3 ± 0.2 vs. HFM 7.0 ± 0.3 cells/area) (NC vs. HF or HFM, p < 0.05; HF vs. HFM, p < 0.05) (Figure 4).

### Miglitol enhanced the gene and protein expressions of UCP1 in BAT of HFM mice

**Figure 5:**
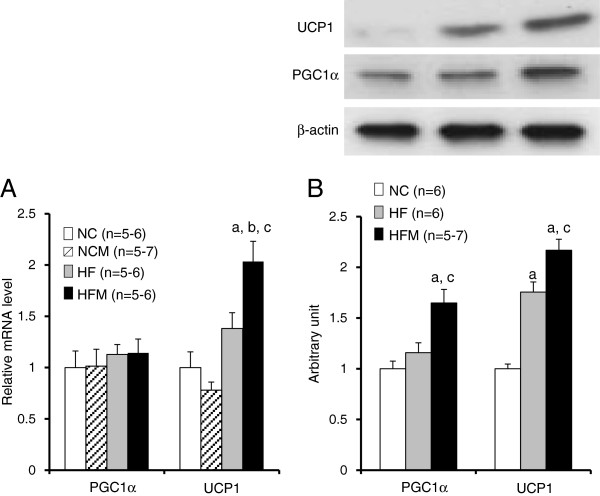
**Effect of miglitol on the expressions of PGC1α and UCP1 in BAT. A**: Real-time PCR experiments. **B**: Western blot analysis. Values are means ± SE for 5–7 mice. ^a^P < 0.05, vs mice fed normal chow diet (NC). ^b^P < 0.05, vs mice fed normal chow diet plus miglitol (NCM). ^c^P < 0.05, vs mice fed high-fat diet alone (HF).

**Figure 6:**
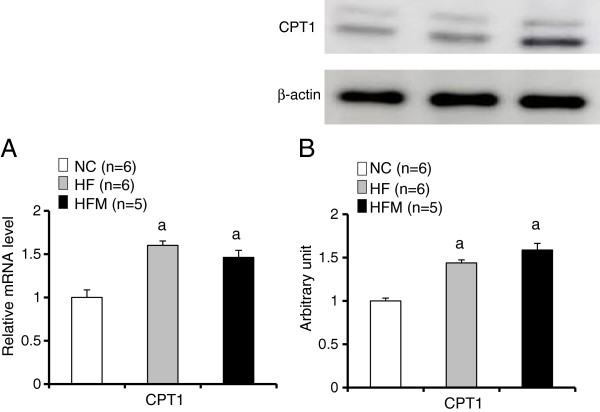
**Effect of miglitol on the expression of CPT1. A**: Real-time PCR experiments. **B**: Western blot analysis. Values are means ± SE for 5–6 mice. ^a^P < 0.05, vs mice fed normal chow diet (NC). ^c^P < 0.05, vs mice fed high-fat diet alone (HF).

The main function of BAT is thermogenesis, which is mediated by upregulation of UCP1. PGC1α is transcriptional coactivator that is required for expression of the UCP1 gene. We evaluated gene and protein expressions of PGC1α and UCP1. The mRNA levels of PGC1α showed no differences between the four groups. However, the level of PGC1α protein of HFM mice was 1.4-fold higher than that of HF mice (p < 0.05) (Figure 5A, B). The expression of UCP1 mRNA in HFM mice was 1.5-fold higher than that of HF mice (p < 0.05). Miglitol did not enhance the expression of UCP1 mRNA in normal chow-fed mice. The level of UCP1 protein in HF mice was 1.7-fold higher than that of control mice (p < 0.05), and the level of UCP-1 protein of HFM mice was 1.2-fold higher than that of HF mice (p < 0.05) (Figure 5A, B). We measured CPT1 expression in BAT to evaluate mitochondrial β-oxidation. The expressions of CPT1 mRNA and protein were significantly increased in both HF mice and HFM mice as compared with control mice. (Figure 6A, B).

### Miglitol enhanced β3-adrenergic signaling in BAT of HFM mice

**Figure 7:**
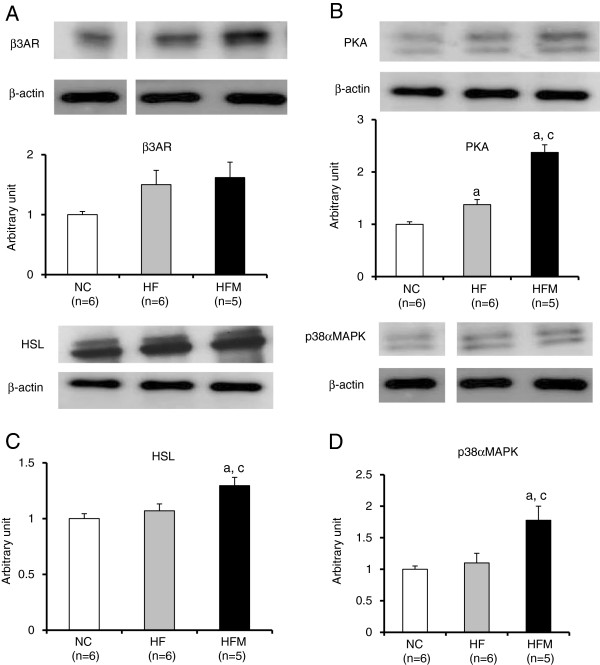
**Effect of miglitol on β3-adrenergic signaling.** Protein expressions of β3-adrenergic signaling in BAT were analyzed. **A-D**: Western blot analysis. Values are means ± SE for 5-6mice. ^a^P < 0.05, vs mice fed normal chow diet (NC). ^c^P < 0.05, vs mice fed high-fat diet alone (HF).

**Figure 8:**
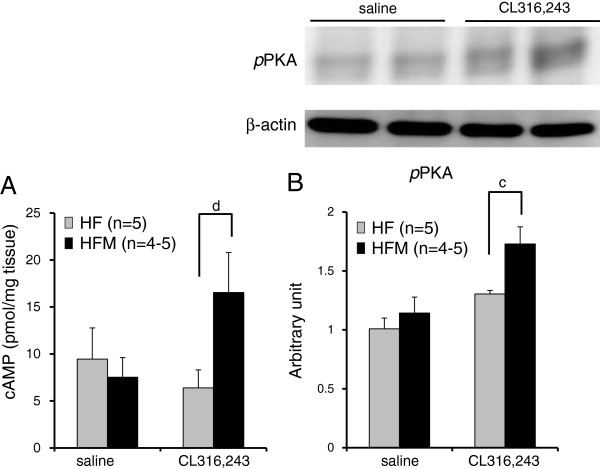
**Effect of miglitol on cAMP and *p*PKA in the presence of a β3-adrenergic receptor agonist. A**: cAMP amount in BAT. **B**: Western blot analysis. Values are means ± SE for 4–5 mice. ^c^P < 0.05, vs mice fed high-fat diet alone (HF). ^d^P < 0.1, vs mice fed high-fat diet alone (HF).

β3-adrenergic signaling through the β3-adrenergic receptor (β3AR) activates UCP1 and thus has a role in reducing obesity. The protein expression of β3AR was not significantly different between HF and HFM mice (Figure 7A). However, the protein expressions of PKA, HSL and p38α MAPK of HFM mice were significantly increased as compared with HF mice (1.7, 1.2 and 1.5-fold, respectively) (p < 0.05) (Figure 7B-D). To test whether miglitol’s upregulation of UCP1 expression was mediated by β3-adrenergic signaling, we measured the effect of a selective β3AR agonist (CL316,243). CL316,243 induced greater amounts of cAMP and *p*PKA protein in HFM mice than in HF mice (Figure 8A, B).

### Hepatic glucokinase expression did not affect thermogenesis in BAT

**Figure 9:**
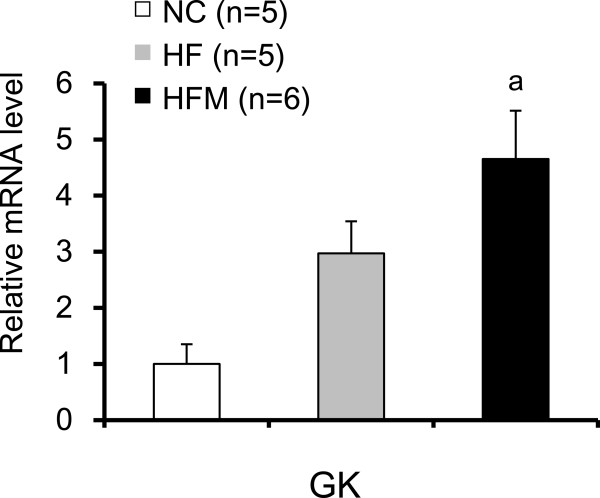
**Effect of miglitol on the gene expression of liver glucokinase (GK).** Real-time PCR experiments. Values are means ± SE for 5-6mice. ^a^P < 0.05, vs mice fed normal chow diet (NC). ^c^P < 0.05, vs mice fed high-fat diet alone (HF).

During the course of this study, it was reported that hepatic glucokinase (GK) expression suppressed thermogenesis in BAT [15]. This raised the possibility that miglitol acts by suppressing liver GK expression. However, miglitol did not suppress GK mRNA expression (Figure 9).

## Discussion

Our data show that miglitol reduced body weight gain and insulin resistance, consistent with a previous study using spontaneous-onset obese type 2 diabetes mice [11]. Recent studies have focused on BAT as a target of treatment for obesity [3,4,16]. We hypothesized that the reason of suppressed body weight gain observed in miglitol-treated mice was the upregulation of energy expenditure. BAT produces heat by non-shivering thermogenesis to maintain body temperature [3]. The key component of this process is UCP1. UCP1, the archetypal UCP, is uniquely expressed in mitochondria of brown adipocytes. UCP1 uncouples adenosine-5′-triphosphate (ATP) synthesis from substrate oxidation in brown adipocytes [17]. When UCP1 is activated, chemical energy is dissipated as heat without ATP synthesis. The upregulation of UCP1 expression indicates increased thermogenesis and energy expenditure, which helps to protect from fat accumulation and obesity [18]. The present study showed that miglitol upregulated UCP1 in BAT of high fat diet-induced obese mice. Consistent with increased UCP1 expression, oxygen consumption was increased in HFM mice (Figures 2 and 5). Miglitol induced an increased interscapular temperature, which can be explained by its stimulation of UCP1 expression in BAT (Figure 3). Brown adipocytes contain a large number of mitochondria and are highly innervated by the sympathetic nervous system (SNS). SNS nerve terminals of BAT release noradrenaline, which activates β-adrenergic receptors and a cascade of events leading to mitochondriogenesis and increased expression of UCP1. Brown adipocytes express various adrenergic receptors that include the α_1_- and β_3_- receptors. β3-adrenergic receptor (β3AR) is, at least in rodents, the main adrenergic receptor in driving the cascade of events necessary for thermogenesis in BAT. β3AR interacts with Gα to stimulate adenylyl cyclase activity, which promotes synthesis of cAMP. Increased sympathetic stimulation induces β3AR activation, increased cAMP generation and subsequent activation of PKA [19]. The protein levels of PKA were higher in HFM mice than in HF mice in our experiment (Figure 7B), which suggests that the upregulation of UCP1 observed in our study involved β3-adrenergic signaling. To confirm that upregulation of UCP1 involves β3-adrenergic signaling, we evaluated the downstream signaling of PKA.

PKA induces lipolysis by activating hormone-sensitive lipase (HSL). HSL releases free fatty acids from intracellular lipid stores [19], which are then transformed into acyl-CoA. Acyl-CoA is combined with carnitine by CPT1 and transported into the mitochondrial matrix as acyl-carnitine. Acyl-carnitine is converted back to acyl-CoA, which can then enter the fatty acid β-oxidation pathway. Free fatty acids not only act as substrates for β-oxidation but also stimulate UCP1 activity [17,19]. The expressions of CPT1 mRNA and protein were not significantly different between HF mice and HFM mice in our study (Figure 6A, B), which suggests that the β-oxidation activity was similar in the two groups. The HFM mice had higher HSL protein levels than the HF mice. The findings that miglitol decreased the number of lipid droplets in BAT cells (Figure 4) and increased the protein expression of HSL (Figure 7C) suggest that lipolysis was activated by miglitol under the high-fat diet. The lipolysis induced by miglitol activated UCP1.

The chronic effects of PKA activation include mitochondrial biogenesis and increased UCP1 gene expression [19]. p38αMAPK is reported to induce UCP1 expression by stimulating the SNS [20-22]. In mouse adipocytes and animal models, β-AR stimulation triggers a kinase cascade from PKA to p38MAPK, which phosphorylates PGC1α [20,21]. PGC1α strongly coactivates several nuclear receptors that bind to the UCP1 enhancer and upregulates UCP1 gene expression [23]. These events also contribute to the orchestrated response to increase mitochondriogenesis and the overall thermogenic capacity of brown adipocytes. The finding that protein levels of p38α MAPK and PGC1α were higher in HFM mice than in HF mice (Figure 5B, Figure 7D) suggests that the gene expression of UCP1 was upregulated through the PKA-p38MAPK-PGC1α cascade by miglitol in high fat diet-induced obese mice.

A β3AR agonist (CL316,243) was found to increase PGC1α mRNA and UCP1 mRNA in 4–6 hours [24,25]. In our study, CL316,243 produced greater amounts of cAMP and *p*PKA protein in HFM mice than in HF mice (Figure 8A, B), confirming that miglitol enhanced β3-adrenergic signaling under the high fat diet.

Glucagon-like peptide 1 (GLP1) is secreted from L cells in the intestine, and promotes insulin secretion in a glucose-dependent manner following ingestion of carbohydrate [26]. GLP1 receptor agonists have been used for the treatment of type 2 diabetes patients in recent years. GLP1 has the potential to be used as an anti-obesity drug [27]. GLP1 not only stimulates insulin secretion but also decreases appetite and reduces food intake when administered either peripherally or directly into the central nerve system [28]. Though miglitol enhances GLP1-secretion in obese humans [29-31], plasma active GLP1 levels in the HF and HFM mice in this study were not significantly different (Table 1), which suggests that GLP1 did not participate in the reduction of obesity in this study.

It remains unclear how miglitol induces thermogenesis in BAT. One possibility is that miglitol stimulates the SNS, which is known to enhance β3-adrenergic signaling [19], which in turn induces thermogenesis in BAT. One way in which miglitol could stimulate the SNS is by suppressing hepatic glucokinase (GK) expression [15]. However, miglitol did not suppress GK mRNA expression in our study (Figure 9).

β3AR agonists increase oxygen consumption and lead to weight loss in obese rodents [32,33]. However, β3AR stimulants have not yet become available, partly because the specificity of β3AR agonists for human β3AR is low [34]. Miglitol has few side effects in humans, and is feasible for long-term use as an oral drug. The adverse effects of miglitol are mainly minor gastrointestinal symptoms. If these side effects are acceptable, miglitol has promise as an anti-obesity drug. If the side effects are not acceptable, another approach is to develop new therapeutics based on the mechanisms of miglitol.

## Conclusions

Miglitol 1) increased energy expenditure, 2) had an anti-obesity effect in high fat diet-induced obese mice, 3) enhanced β3-adrenergic signaling and 4) upregulated UCP1 in BAT under the condition of a high fat diet. These findings suggest that miglitol has the potential to be a therapeutic for the treatment of obesity.

## Abbreviations

αGI: Alpha-glucosidase inhibitor; BAT: Brown adipose tissue; VO2: Oxygen consumption; HOMA-R: Homeostasis model assessment of insulin resistance; GLP1: Glucagon-like peptide1; UCP1: Uncoupling protein 1; PGC1α: Peroxisome proliferator-activated receptor gamma coactivator 1α; β3AR: β3-adrenergic receptor; PKA: Protein kinase A; pPKA: Phosphorylated protein kinase A; HSL: Hormone -sensitive lipase; p38αMAPK: p38 α mitogen-activated protein kinase; CPT1: Carnitine palmitoyltransferase I; GK: Glucokinase; SNS: Sympathetic nervous system; NC group: Mice fed normal chow diet alone; NCM group: Mice fed a normal chow diet plus miglitol; HF group: Mice fed high fat diet alone; HFM group: Mice fed a high fat diet plus miglitol.

## Competing interests

The authors have no competing interests.

## Authors’ contributions

SS, HN and KK conceived the study and designed the experimental plan. SS performed all of the experiments and contributed to data collection. SS and HN contributed to data analysis, interpretation and manuscript writing. JM contributed to data interpretation and manuscript writing. KK, KM and WA contributed to data collection. KY, HI and HH contributed to data interpretation. All authors read and approved the final manuscript.
